# CheekAge, a next-generation epigenetic buccal clock, is predictive of mortality in human blood

**DOI:** 10.3389/fragi.2024.1460360

**Published:** 2024-10-01

**Authors:** Maxim N. Shokhirev, Daniel J. Kramer, Janie Corley, Simon R. Cox, Trinna L. Cuellar, Adiv A. Johnson

**Affiliations:** ^1^ Tally Health, New York, NY, United States; ^2^ Lothian Birth Cohorts, Department of Psychology, University of Edinburgh, Edinburgh, United Kingdom

**Keywords:** DNA methylation, mortality, epigenetic aging clock, biomarker, aging, longitudinal

## Abstract

While earlier first-generation epigenetic aging clocks were trained to estimate chronological age as accurately as possible, more recent next-generation clocks incorporate DNA methylation information more pertinent to health, lifestyle, and/or outcomes. Recently, we produced a non-invasive next-generation epigenetic clock trained using Infinium MethylationEPIC data from more than 8,000 diverse adult buccal samples. While this clock correlated with various health, lifestyle, and disease factors, we did not assess its ability to capture mortality. To address this gap, we applied CheekAge to the longitudinal Lothian Birth Cohorts of 1921 and 1936. Despite missing nearly half of its CpG inputs, CheekAge was significantly associated with mortality in this longitudinal blood dataset. Specifically, a change in one standard deviation corresponded to a hazard ratio (HR) of 1.21 (FDR *q* = 1.66e-6). CheekAge performed better than all first-generation clocks tested and displayed a comparable HR to the next-generation, blood-trained DNAm PhenoAge clock (HR = 1.23, *q* = 2.45e-9). To better understand the relative importance of each CheekAge input in blood, we iteratively removed each clock CpG and re-calculated the overall mortality association. The most significant effect came from omitting the CpG cg14386193, which is annotated to the gene *ALPK2*. Excluding this DNA methylation site increased the FDR value by nearly threefold (to 4.92e-06). We additionally performed enrichment analyses of the top annotated CpGs that impact mortality to better understand their associated biology. Taken together, we provide important validation for CheekAge and highlight novel CpGs that underlie a newly identified mortality association.

## Introduction

Machine learning models that predict age using DNA methylation information are referred to as epigenetic aging clocks. Their output, which is often described as epigenetic age or DNA methylation age, represents a unique, more contemporary aging biomarker. These epigenetic clocks can be trained for a variety of tasks, such as estimating chronological age or predicting health outcomes in a population (Bell et al., 2019). Thus, they have value across multiple lines of inquiry, including forensics and biogerontology. First-generation clocks are simply trained to predict chronological age and are more relevant to forensics. Conversely, next-generation clocks are optimized to incorporate methylation information linked to health, lifestyle, and/or outcomes.

The majority of next-generation clocks require blood collection, which can be challenging to perform in a home setting and in older adults. To address this, we recently created the next-generation clock CheekAge using a large Infinium MethylationEPIC buccal dataset spanning more than 8,000 adults (Shokhirev et al., 2024). This model, which utilizes more than 200,000 DNA methylation sites to produce an epigenetic age estimate in an easy-to-collect tissue, was trained to correlate with a variety of lifestyle and health factors, including weekly exercise, sleep quality, diet, stress, smoking status, alcohol intake, and body mass index. For example, health-promoting behaviors like more frequent weekly exercise were associated with a lower delta age (epigenetic age – chronological age) while less health-promoting behaviors like heavy alcohol intake correlated with a higher delta age. In addition to correlating with chronological age in external datasets, CheekAge was significantly elevated in patients with progeria, meningioma, or respiratory infections as well as in childhood cancer survivors who had undergone radiation therapy (Shokhirev et al., 2024). However, CheekAge’s ability to estimate mortality has not yet been assessed. Since an ideal aging biomarker is one that can capture mortality risk in a longitudinal setting, we sought to evaluate the ability of this buccal clock to predict the risk of death.

## Methods and materials

The Lothian Birth Cohorts (LBC) research group is based in the department of Psychology at the University of Edinburgh. The core purpose is to understand cognitive and brain aging and their determinants including metrics such as lifestyle and psychosocial factors as well as biomedical, genetic, epigenetic, and brain imaging data. The cohorts comprise two longitudinal studies, one starting with older individuals born in 1921 and a second with individuals born in 1936 (Deary et al., 2012; Taylor et al., 2018). Participants are tested every 3 years. In order to test whether delta CheekAge was significantly associated with mortality risk, we obtained access to the Infinium HumanMethylation450 and phenotypic data from both cohorts. Raw methylation data was processed using the *minfi* package v1.46.0 as described in detail previously (Shokhirev et al., 2024). Since our clock was trained on Infinium MethylationEPIC array buccal data, the Infinium HumanMethylation450 array data was missing roughly half of the inputs used to calculate CheekAge. To use the CheekAge clock on the Infinium HumanMethylation450 data, we removed all missing inputs from the cluster averaging process and clusters with no CpG inputs present were set to 0. This resulted in surprisingly little loss of accuracy when testing in our previously described internal buccal data (Shokhirev et al., 2024), so we were encouraged to apply the same process to the Infinium HumanMethylation450 blood data from the LBC. We then normalized by dividing the resulting delta ages by the standard deviation of the delta ages to obtain standardized 450 k delta CheekAge predictions (s 450 k ΔCheekAge).

From there, we fit a Cox Regression Model to estimate the associations of s 450 k ΔCheekAge and other confounding variables on the survival function using the *coxph* function from the survival package (v 3.5-3):

$$Surv\left(Age,Status\right)∼s\;450k\;\Delta CheekAge+N+L+M+NI+Sex+Age+Cohort+Timepoint$$

where $Surv\left(Age,Status\right)$ is the survival function and status is alive (0), or deceased (1) at last measurement, s 450k ΔCheekAge is the standardized delta CheekAge at last measurement, and N, L, M, NI, Sex, Age, Cohort, and Time point are neutrophil proportion, lymphocyte proportion, monocyte proportion, non-immune cell type proportion, predicted sex, chronological age, LBC cohort, and LBC wave, respectively. Additional epigenetic aging clocks were similarly analyzed using the R methylCIPHER package (Thrush et al., 2022).

We then systematically set each of the 115,553 CpGs overlapping with the CheekAge clock to zero, and recalculated the Cox Regression Model using the updated s 450k ΔCheekAge for each CpG, to reveal the effect (defined as the magnitude of the statistical change between base and removed/set-to-zero models) of each CpG on mortality association in the normalized CheekAge clock. Gene annotations for the CpGs with the largest effect on mortality association were manually reviewed. The cell type proportions were predicted using the *EpiDISH* package v2.16 hepidish function with ref1.m = centEpiFibIC.m, ref2.m = centBloodSub.m, h.CT.idx = 3 (Zheng et al., 2019). We defined the lymphocyte proportion as the sum of the CD8T, B, and CD4T cell proportions, and the non-immune proportion as the sum of the fibroblast and epithelial cell proportions. The sex was predicted using the *minfi* package v1.46.0, getSex function (Aryee et al., 2014). The forest plot was generated using *ggforest* and the adjusted survival curves were generated using the *ggadjustedcurves* function with method “marginal” from the survminer package (v 0.4.9). Adjusted *p*-values were calculated using the p.adjust R function with the “fdr” method.

Similarly to before (Shokhirev et al., 2024), we used WebGestalt (Elizarraras et al., 2024) to perform network topology-based enrichment analyses of annotated CpGs that had the most dramatic impact on the overall mortality association. The network used was PPI BioGrid (Oughtred et al., 2021), the set number of top ranking neighbors was equivalent to 125 (half the input size), and the significance level cut-off was set to an FDR of 0.05. For the Gene Ontology (Gene Ontology et al., 2023) results that were returned, a category size cut-off of 1,000 was put in place and a weight set cover algorithm was employed to reduce redundancy to the top 15 categories. We specifically used the WebGestaltR package (https://cran.r-project.org/package=WebGestaltR).

## Results

To further investigate CheekAge, we turned to the longitudinal mortality data from the LBC of 1921 and 1936 (LBC1921 and LBC1936) (Deary et al., 2012; Taylor et al., 2018). These two studies of community-dwelling older adults comprise 1,513 participants (712 males and 801 females) with methylation data who were monitored in four different waves. Mortality status was derived based on dates of death, identified via data linkage from the National Health Service Central Register, provided by the National Record of Scotland. These were converted to age in days at death by the LBC team and used as the outcome variable. Looking at all four waves, chronological age varied from 67.8 to 90.6 years. The censor dates were January 2022 and September 2023 for the LBC1921 and LBC1936 cohorts, respectively. Cohort characteristics, including age, sex, and number of subjects for each wave, are provided in Supplementary Table 1.

**FIGURE 1:**
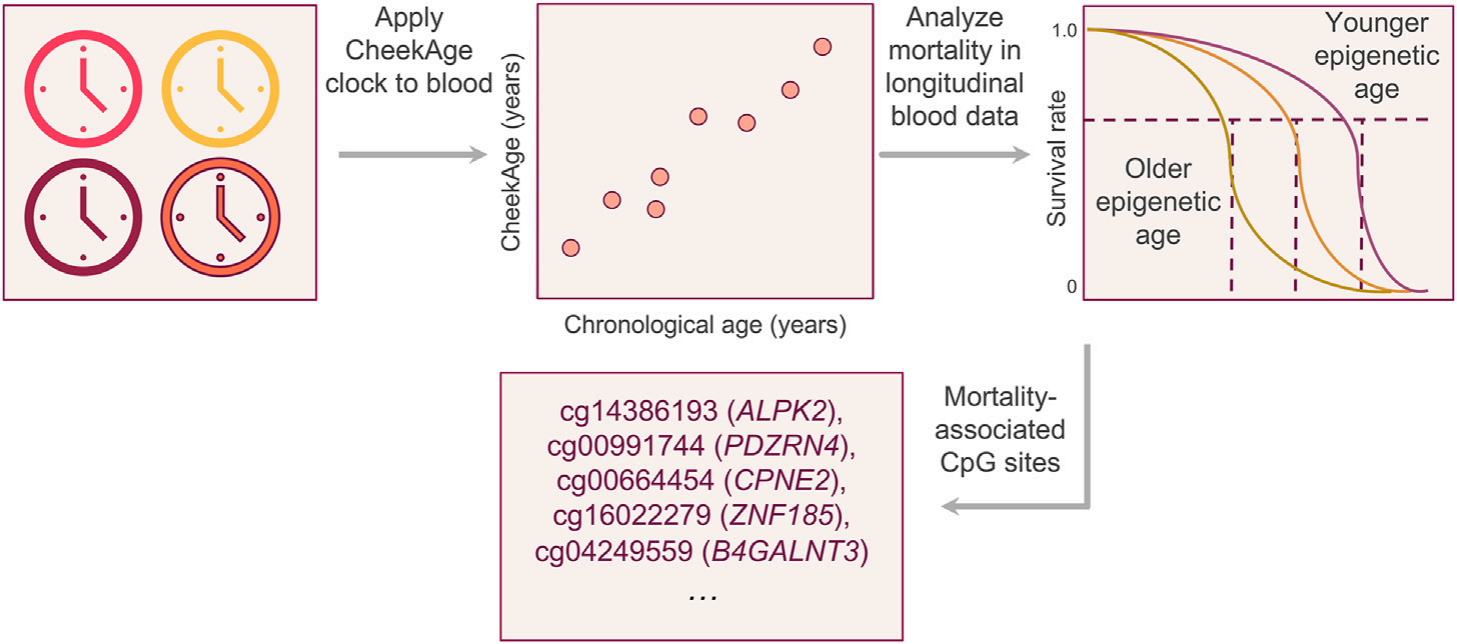
Visual summary of the experimental approach. After demonstrating that the buccal clock CheekAge can be applied to Infinium HumanMethylation450 blood methylation data, we analyzed the relationship between mortality risk and the disparity between CheekAge and chronological age in the longitudinal Lothian Birth Cohorts. After identifying a significant relationship, we iteratively removed each CpG from the model and re-calculated the significance of the mortality association to better understand the relationship between each input and mortality.

**FIGURE 2:**
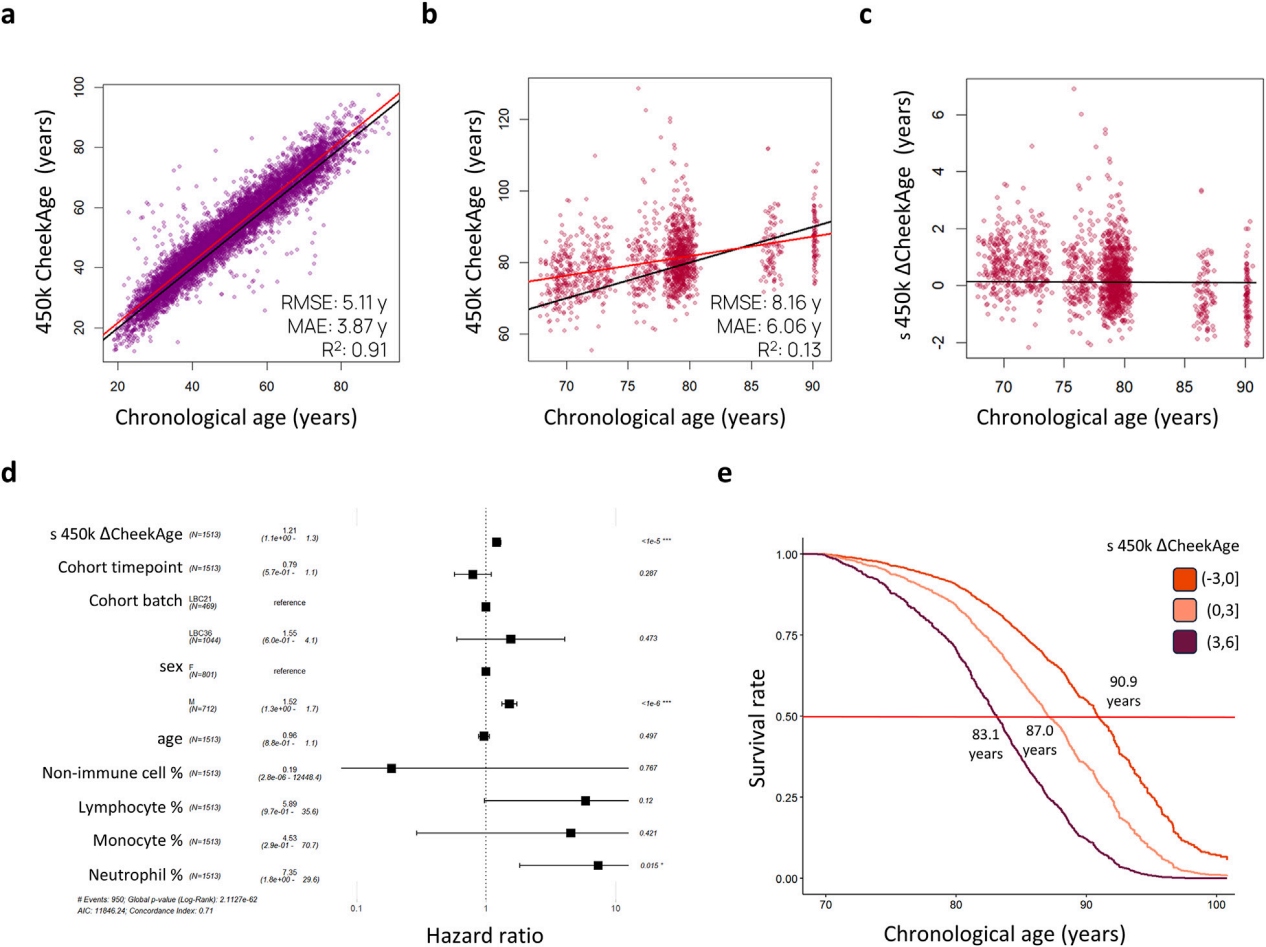
Standardized delta CheekAge is significantly associated with mortality in the Lothian Birth Cohorts. **(A)** CheekAge predictions on internal buccal data using only CpGs found on Infinium HumanMethylation450 arrays. **(B)** CheekAge predictions on blood data from the Lothian Birth Cohorts. **(C)** Scatterplot showing standardized delta CheekAge as a function of chronological age. Since the scale for CheekAge is arbitrary, delta CheekAge was normalized by dividing by the standard deviation. **(D)** Forest plot showing hazard ratios for the Cox regression model. Each row shows the variable, number of observations, hazard ratio, 95% CI, and FDR adjusted significance of association. **(E)** Marginal survival curves for three categories of delta CheekAge: −3 standard deviations to 0, 0–3 standard deviations, and 3–6 standard deviations. Labels indicate chronological age at a 50% survival rate (middle red line). In the scatterplots **(A–C)**, black lines indicate y = x or y = 0 lines and red lines indicate best fit lines. RMSE and MAE in the first two scatterplots **(A, B)** stand for root mean squared error and mean absolute error, respectively.

As visualized in Figure 1, our aim was to determine whether or not CheekAge significantly associates with mortality in LBC data and, if so, identify which specific DNA methylation sites are driving the mortality association. We began by predicting CheekAge using the final methylation data measured in participants prior to their death or censor date. Since the LBC data were measured using Infinium HumanMethylation450 arrays, roughly half of the CpGs used by CheekAge were not measured. We first evaluated the effect of using available Infinium HumanMethylation450 CpGs to predict CheekAge in our previously described methylomic buccal data derived from more than 8,000 diverse adults (51.9% male and 48.1% female) spanning a chronological age range of 18–93 years (Shokhirev et al., 2024). While the R^2^ and mean absolute error (MAE) values of the full CheekAge model (using all available CpGs) were respectively 0.93 and 3.05 years (Shokhirev et al., 2024), the R^2^ was 0.91 and the MAE was 3.87 years in the current model using only Infinium HumanMethylation450 CpGs (Figure 2A). Encouraged by this minimal loss in accuracy, we next predicted CheekAge in the LBC data directly (Figure 2B) and then standardized the delta age by dividing the delta age by the standard deviation of all delta ages (Figure 2C).

**FIGURE 3:**
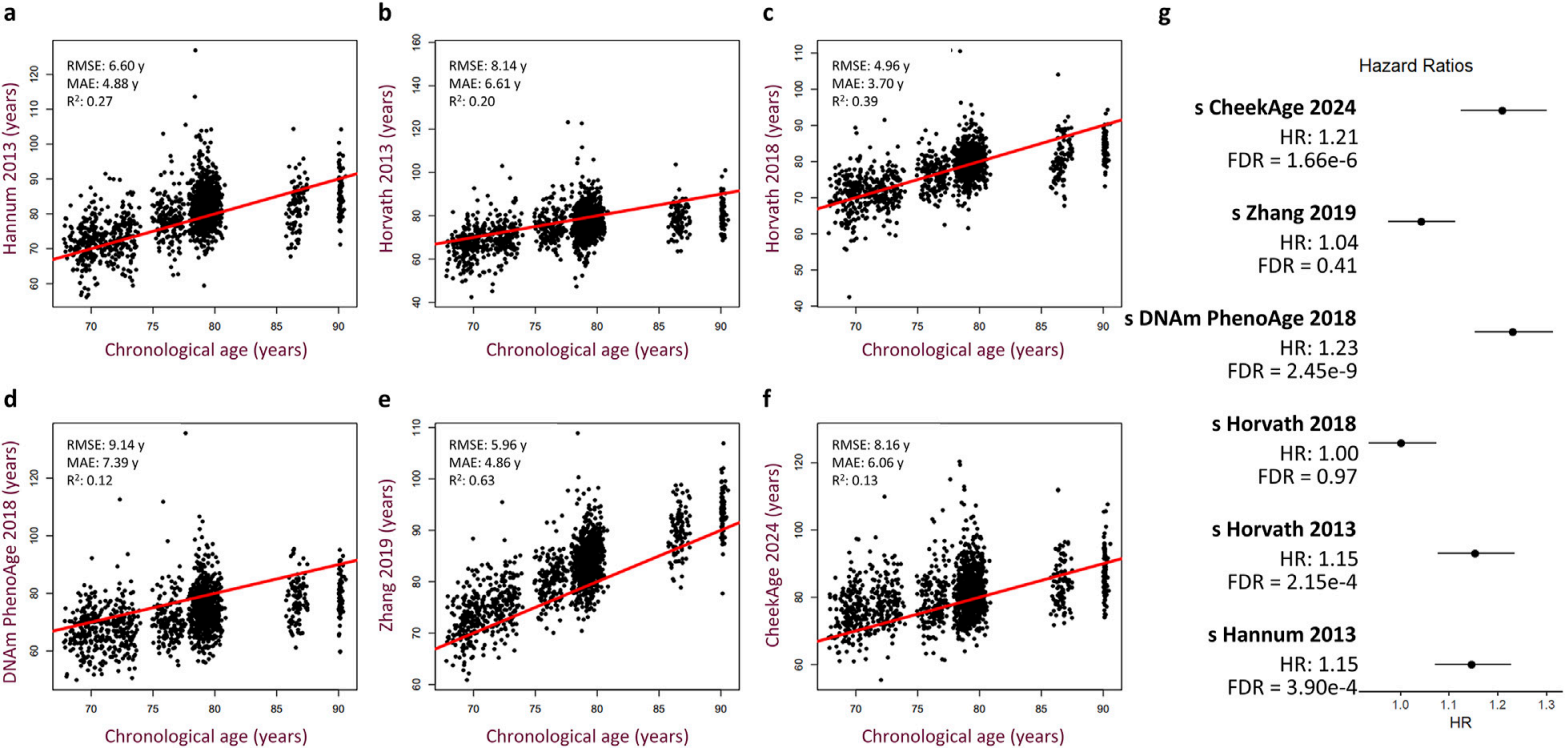
Performance of other published epigenetic age clocks in the Lothian Birth Cohorts. **(A–F)** Scatterplots showing predicted age versus chronological age and best fit linear regression lines for the Hannum 2013 **(A)**, Horvath 2013 **(B)**, Horvath 2018 **(C)**, DNAm PhenoAge 2018 **(D)**, Zhang 2019 **(E)**, and CheekAge 2024 **(F)** clocks. Root mean squared error (RMSE), mean absolute error (MAE), and squared Pearson correlation coefficient (R^2^) values are shown for each clock. **(G)** Hazard ratios with 95% CI and FDR values are shown for each clock. Cox regression models include delta age, chronological age, timepoint, cohort, sex, and cell type composition as covariates.

To test for a significant association between this epigenetic clock and mortality risk, we trained a Cox proportional hazards regression model taking into account sex, age, cohort, time point of last measurement, cell type proportions, and standardized delta CheekAge (s 450 k ΔCheekAge). We demonstrate that s 450 k ΔCheekAge has a significant hazard ratio (HR) of 1.21 for each standard deviation with an adjusted false discovery rate (FDR) of 1.66e-6 (Figure 2D). Compared to the −3 to 0 standard deviation group, the HR for the three to 6 standard deviation group was 2.48 (FDR = 0.004). To demonstrate the effect on survival, we show the survival curves for three groups stratified by s 450 k ΔCheekAge, with the lowest s 450 k ΔCheekAge group expected to reach 50% survival 7.8 years after the highest s 450 k ΔCheekAge group (Figure 2E). To better contextualize the relative significance of this association, we looked at the ability of five other epigenetic aging clocks to capture mortality in the LBC data (Figure 3). Specifically, we looked at the Hannum 2013, Horvath 2013, Horvath 2018, DNAm PhenoAge (Levine et al., 2018), and Zhang 2019 clocks. DNAm PhenoAge represents a next-generation model while the other epigenetic biomarkers (Hannum et al., 2013; Horvath, 2013; Horvath et al., 2018; Zhang et al., 2019) are first-generation clocks. Scatterplots (Figures 3A–F) and mortality association statistics (Figure 3G) are shown for each clock. The Horvath 2018 (HR = 1.00, FDR = 0.97) and Zhang 2019 (HR = 1.04, FDR = 0.41) showed non-significant mortality associations while the Hannum 2013 (HR = 1.15, FDR = 3.90e-4) and Horvath 2013 (HR = 1.15, FDR = 2.15e-4) displayed mortality associations that were significant, albeit less so compared to CheekAge. The HR for the blood-trained, next-generation clock DNAm PhenoAge (HR = 1.23, FDR = 2.45e-9) was comparable to CheekAge and in line with what has been previously reported (Stevenson et al., 2019). These analyses suggest that, even with limited CpG inputs collected in a different tissue, CheekAge is significantly associated with mortality in a longitudinal dataset and outcompetes first-generation clocks trained in datasets containing blood data. All of the CpGs included in the s 450 k ΔCheekAge model as well as DNA methylation sites that overlap between our model and externally tested clocks are listed in Supplementary Table 2. Interestingly, the CpG cg19722847 (annotated to the gene *IPO8*) was shared across the CheekAge, Hannum 2013, Horvath 2013, Horvath 2018, DNAm PhenoAge, and Zhang 2019 clocks. Not only do zebrafish lacking the gene annotated to this CpG display skeletal and cardiovascular defects, but human loss of function mutations in *IPO8* underlie a connective tissue disorder characterized by immune dysfunction as well as skeletal and cardiovascular anomalies (Ziegler et al., 2021).

To better understand which CpGs have the biggest effect on the association between CheekAge and mortality, we systematically removed each of the 115,553 CpGs overlapping with the CheekAge clock model in the LBC Infinium HumanMethylation450 methylation data and recalculated the adjusted FDR of the mortality association. If removing a CpG made the mortality prediction less significant, it was presumed to play a role in driving the mortality association and dubbed a mortality CpG. Conversely, we refer to CpGs whose removal made the mortality prediction more significant as anti-mortality CpGs. The distribution of mortality and anti-mortality CpGs, including their effect on FDR, is visually summarized in Supplementary Figure S1. When we sort these 115,553 CpGs (genes annotated to each CpG are indicated by parentheses) by impact (Supplementary Table 2), the 10 CpGs that most dramatically impair the clock’s mortality association when removed are cg14386193 (*ALPK2*), cg00991744 (*PDZRN4*), cg00664454 (*CPNE2*), cg16022279 (*ZNF185*), cg04249559 (*B4GALNT3*), cg20313963 (*SLC2A3*), cg18170680, cg18154784 (*SAT1*), cg09936008 (*ZNF213*), and cg20210051 (*PDZRN4*). It is interesting to note that, among this top 10 list, two CpGs (cg00991744 and cg20210051) are annotated to the putative tumor suppressor gene *PDZRN4* (Lu et al., 2019; Jin et al., 2022). The CpG whose removal most prominently attenuated the model’s mortality association was cg14386193 (*ALPK2*). Excluding this DNA methylation site from the model increased the FDR value by approximately threefold, from 1.66e-06 (full model using all available CpGs) to 4.92e-06 (model lacking cg14386193). This CpG is annotated to the gene *ALPK2*, which encodes for Alpha-protein kinase 2 and is highly expressed in fibroblasts, heart, and muscle relative to other tissues in the body (https://www.gtexportal.org/home/gene/ENSG00000198796) according to the Genotype-Tissue Expression (GTEx) project (Consortium, 2013). Interestingly, this gene is upregulated in human bladder cancer and knocking down *ALPK2* in a mouse xenograft model of bladder cancer suppressed tumor growth (Wang et al., 2021). The gene *alpk2* was found to be essential for proper cardiac development and function in zebrafish (Hofsteen et al., 2018), though cardiac function and morphology were normal in mice lacking *Alpk2* (Bogomolovas et al., 2020). Separately, *Alpk2* was implicated as a potential contributor to genetic hypertension in the Dahl salt-sensitive rat model (Chauvet et al., 2011). It would be intriguing to determine if the manipulation of this gene impacts lifespan in animal models.

For each annotated gene connected to these top 10 CpGs, an interesting literature connection germane to aging and/or age-related disease is provided in Table 1. Collectively, these literature connections are pertinent to survival, cancer, osteoporosis, inflammation, and metabolic syndrome. Two that are especially worth highlighting are cg04249559 (*B4GALNT3*) and cg18154784 (*SAT1*), the former of which was previously reported to be associated with all-cause mortality in the LBC (Lund et al., 2019). Excluding cg04249559 (*B4GALNT3*) or cg18154784 (*SAT1*) from the model respectively raised the FDR to 2.72e-06 (1.6-fold increase) or 2.43e-06 (1.5-fold increase). In mice, knocking out *B4galnt3* (which encodes Beta-1,4-N-acetylgalactosaminyltransferase 3) decreases bone mass and elevates circulating levels of sclerostin, a small protein that can be inhibited to reduce fracture risk. In humans, a Mendelian randomization analysis found a causal association between *B4GALNT3* variants and a higher risk of fractures and lower bone mineral density (Moverare-Skrtic et al., 2023). The CpG site cg18154784 is found in the 3′UTR region of the X-chromosome-located gene *SAT1. SAT1* encodes for Diamine acetyltransferase 1, an enzyme that regulates the metabolism of spermine and spermidine via acetylation (Pegg, 2008). Tissue expression analysis by GTEx suggests that it is highly expressed in minor salivary gland tissue (https://www.gtexportal.org/home/gene/ENSG00000130066). In a mouse model of diet-induced obesity, chemically activating Sat1 via the synthetic agent triethylenetetramine dihydrochloride stimulated autophagy, reduced weight, and improved both fatty liver and glucose intolerance (Castoldi et al., 2020). Per the EWAS Data Hub (Xiong et al., 2020), decreased methylation at this site has been reported in Parkinson’s disease brain samples (https://ngdc.cncb.ac.cn/ewas/datahub/probe/cg18154784). Moreover, the polyamine pathway has been previously implicated in the pathogenesis of Parkinson’s disease (Lewandowski et al., 2010). Spermidine, which is regulated by *SAT1*, has also been reported to induce autophagy and extend lifespan in multiple animal models (Hofer et al., 2022). Future investigations are warranted to determine if the expression of these genes are impacted by these mortality-linked CpG sites. Such work, for example, has been done to demonstrate that the hypermethylation of *Elovl2* decreases gene expression and drives age-related visual dysfunction in mice (Chen et al., 2020).

**TABLE 1:** The 10 CpGs that most markedly reduced the significance of the mortality association when removed from the full model. For each CpG, the FDR for the model lacking that CpG is provided. For each gene annotation, the full name for the protein encoded by that gene and an interesting literature connection are provided.

CpG	FDR	Annotated gene	Full name	Interesting literature connection
cg14386193	4.92E-06	ALPK2	Alpha-protein kinase 2	In a mouse xenograft model of bladder cancer, suppressing *ALPK2* in injected cells suppresses tumor formation (Wang et al., 2021)
cg00991744	3.53E-06	PDZRN4	PDZ domain-containing RING finger protein 4	Inhibiting *PDZRN4* in human prostate cancer cells increases tumor weight in nude mice (Jin et al., 2022)
cg00664454	3.52E-06	CPNE2	Copine-2	*CPNE2* is underexpressed in glioblastoma patient-derived glial cells overexpressing the tumor suppressor *WWOX* (Kaluzinska-Kolat et al., 2023)
cg16022279	2.75E-06	ZNF185	Zinc finger protein 185	In response to inflammatory stimuli, mice lacking *Zfp185* (mouse ortholog of *ZNF185*) display higher amounts of vascular leakage (Suzuki et al., 2023)
cg04249559	2.72E-06	B4GALNT3	Beta-1,4-N-acetylgalactosaminyltransferase 3	Bone mass is reduced in mice lacking *B4galnt3* and, in humans, *B4GALNT3* variants causally associate with a lower bone mineral density and higher fracture risk (Moverare-Skrtic et al., 2023)
cg20313963	2.60E-06	SLC2A3	Solute carrier family 2, facilitated glucose transporter member 3	Neuronal deletion of *Slc2a3* markedly decreases survival in mice (Shin et al., 2018)
cg18170680	2.47E-06	N/A	N/A	N/A
cg18154784	2.43E-06	SAT1	Diamine acetyltransferase 1	In a mouse model of diet-induced obesity, chemical activation of Sat1 increases autophagy as well as decreases obesity, hepatosteatosis, and glucose intolerance (Castoldi et al., 2020)
cg09936008	2.41E-06	ZNF213	Zinc finger protein 213	The expression of *ZNF213* in breast tissue associates with longer relapse survival in patients with triple-negative breast cancer (Liu et al., 2021)
cg20210051	2.39E-06	PDZRN4	PDZ domain-containing RING finger protein 4	In a xenograft cancer model, knocking down *PDZRN4* in breast cancer cells exacerbates tumor growth and metastasis (Lu et al., 2019)

If we expand beyond these top 10 CpGs and look at the top 100 mortality CpGs (Supplementary Table 2), several DNA methylation sites are annotated to genes with tangible connections to lifespan and/or age-related disease. As an example of this, the top 100 CpG *cg25163611* is annotated to *IGF1*, which encodes for Insulin-like growth factor I. Insulin/insulin-like growth factor I signaling represents a canonical, evolutionarily conserved pathway that modulates longevity in model organisms (Singh et al., 2019). Similarly, the top 100 CpG *cg15826479* is annotated to *RPTOR*, which encodes for Regulatory-associated protein of mTOR. Not only does mTOR signaling also represent a canonical longevity pathway in model organisms (Singh et al., 2019), but the neuronal knockdown of *daf-15* (*Caenorhabditis elegans* ortholog of *RPTOR*) extends lifespan and improves age-related health in worms (Zang et al., 2024). Yet another example worth highlighting is the top 100 CpG *cg05433642*, which is annotated to the gene *MBNL2*. This gene encodes for Muscleblind-like protein 2, a regulator of pre-mRNA alternative splicing. Suggestive of an important role relevant to age-related disease, a recent study showed that *Mbnl2* levels accumulate with age in the rat heart and that the inhibition of *Mbnl2* decelerates cardiac fibrosis in aging rats (Lu et al., 2024).

Finally, we used WebGestalt (Elizarraras et al., 2024) to perform network topology-based enrichment analyses of the top 250 annotated mortality CpGs (Supplementary Figure S2) and the top 250 annotated anti-mortality CpGs (Supplementary Figure S3). For each set of top annotated genes, the 15 most significant Gene Ontology (Gene Ontology et al., 2023) processes are shown (Supplementary Figures S2, S3). The 15 most significant processes for mortality CpGs–DNA methylation sites whose removal made the mortality association less significant and were therefore driving significant association with mortality–were as follows: “response to organonitrogen compound,” “post-translational protein modification,” “negative regulation of developmental process,” “regulation of catabolic process,” “rhythmic process,” “regulation of cell cycle,” “response to organic cyclic compound,” “regulation of cellular localization,” “trans-synaptic signaling,” “import into cell,” “apoptotic signaling pathway,” “enzyme-linked receptor protein signaling pathway,” “head development,” “positive regulation of intracellular signal transduction,” and “protein localization to organelle.” These results can be grouped into themes of proteostasis (“post-translational protein modification” and “protein localization to organelle”), cell signaling (“trans-synaptic signaling,” “apoptotic signaling pathway,” “enzyme-linked receptor protein signaling pathway,” and “positive regulation of intracellular signal transduction”), cellular responses (“response to organonitrogen compound” and “response to organic cyclic compound”), and development (“negative regulation of developmental process” and “head development”). The two proteostasis-relevant terms are germane to the established aging hallmark loss of proteostasis (Lopez-Otin et al., 2023).

Turning to the top 250 annotated anti-mortality CpGs (Supplementary Figure S3), the 15 most significant Gene Ontology processes were “gliogenesis,” “response to organonitrogen compound,” “regulation of cell cycle,” “regulation of anatomical structure morphogenesis,” “activation of immune response,” “cell junction organization,” “regulation of membrane potential,” “animal organ morphogenesis,” “synaptic signaling,” “negative regulation of developmental process,” “protein catabolic process,” “negative regulation of multicellular organismal process,” “protein modification by small protein conjugation,” “central nervous system development,” and “supramolecular fiber organization.” It is interesting to note that, for these genes linked to DNA methylation sites that hinder the clock’s mortality association, development and morphogenesis was such a prominent theme (i.e., “regulation of anatomical structure morphogenesis,” “animal organ morphogenesis,” “negative regulation of developmental process,” and “central nervous system development”). In addition, the results “protein catabolic process” and “protein modification by small protein conjugation” suggest a smaller theme of proteostasis. The remaining top processes are eclectic and disparate enough that they’re difficult to group into coherent motifs.

## Discussion

To our knowledge, this is the first study to demonstrate that an aging biomarker optimized for buccal tissue can be applied to blood for mortality prediction. Our findings build on previous work by Lowe et al. (2013) from more than a decade ago, which found that buccal methylation data was highly informative for a variety of phenotypes and diseases. The magnitude of the HR for mortality prediction outcompetes all first-generation clocks tested and compares favorably to the next-generation blood-trained clock DNAm PhenoAge. These data suggest that adult buccal tissue, which is relatively painless and easy to collect in a variety of settings, may represent a rich source of aging biomarkers. Furthermore, it is encouraging that an Infinium MethylationEPIC clock trained in buccal tissue can capture mortality risk in Infinium HumanMethylation450 blood data. Because most methylation changes that occur with age are tissue-specific (Slieker et al., 2018), we hypothesize that the mortality association would be stronger in a longitudinal dataset containing either cheek swab or saliva methylation data. In summary, this work provides further evidence that CheekAge is a next-generation model and reveals novel CpGs linked to human mortality.

## Data Availability

The data analyzed in this study is subject to the following licenses/restrictions: Access to the LBC dataset is application-only. Requests for LBC data access and collaboration can be made via the following link: https://lothian-birth-cohorts.ed.ac.uk/data-access-collaboration.

## References

[B1] Aryee M. J. Jaffe A. E. Corrada-Bravo H. Ladd-Acosta C. Feinberg A. P. Hansen K. D. (2014). Minfi: a flexible and comprehensive Bioconductor package for the analysis of Infinium DNA methylation microarrays. Bioinformatics 30 (10), 1363–1369. 10.1093/bioinformatics/btu049 24478339 PMC4016708

[B2] Bell C. G. Lowe R. Adams P. D. Baccarelli A. A. Beck S. Bell J. T. (2019). DNA methylation aging clocks: challenges and recommendations. Genome Biol. 20 (1), 249. 10.1186/s13059-019-1824-y 31767039 PMC6876109

[B3] Bogomolovas J. Feng W. Yu M. D. Huang S. Zhang L. Trexler C. (2020). Atypical ALPK2 kinase is not essential for cardiac development and function. Am. J. Physiol. Heart Circ. Physiol. 318 (6), H1509–H1515. 10.1152/ajpheart.00249.2020 32383995 PMC7311700

[B4] Castoldi F. Hyvonen M. T. Durand S. Aprahamian F. Sauvat A. Malik S. A. (2020). Chemical activation of SAT1 corrects diet-induced metabolic syndrome. Cell Death Differ. 27 (10), 2904–2920. 10.1038/s41418-020-0550-z 32376874 PMC7494776

[B5] Chauvet C. Crespo K. Menard A. Wu Y. Xiao C. Blain M. (2011). α-Kinase 2 is a novel candidate gene for inherited hypertension in Dahl rats. J. Hypertens. 29 (7), 1320–1326. 10.1097/HJH.0b013e32834705e4 21602714

[B6] Chen D. Chao D. L. Rocha L. Kolar M. Nguyen Huu V. A. Krawczyk M. (2020). The lipid elongation enzyme ELOVL2 is a molecular regulator of aging in the retina. Aging Cell 19 (2), e13100. 10.1111/acel.13100 31943697 PMC6996962

[B7] Consortium G. T. (2013). The genotype-tissue expression (GTEx) project. Nat. Genet. 45 (6), 580–585. 10.1038/ng.2653 23715323 PMC4010069

[B8] Deary I. J. Gow A. J. Pattie A. Starr J. M. (2012). Cohort profile: the lothian Birth cohorts of 1921 and 1936. Int. J. Epidemiol. 41 (6), 1576–1584. 10.1093/ije/dyr197 22253310

[B9] Elizarraras J. M. Liao Y. Shi Z. Zhu Q. Pico A. R. Zhang B. (2024). WebGestalt 2024: faster gene set analysis and new support for metabolomics and multi-omics. Nucleic Acids Res. 52 (W1), W415–W421. 10.1093/nar/gkae456 38808672 PMC11223849

[B10] Gene Ontology C. Aleksander S. A. Balhoff J. Carbon S. Cherry J. M. Drabkin H. J. (2023). The gene Ontology knowledgebase in 2023. Genetics 224 (1), iyad031. 10.1093/genetics/iyad031 36866529 PMC10158837

[B11] Hannum G. Guinney J. Zhao L. Zhang L. Hughes G. Sadda S. (2013). Genome-wide methylation profiles reveal quantitative views of human aging rates. Mol. Cell 49 (2), 359–367. 10.1016/j.molcel.2012.10.016 23177740 PMC3780611

[B12] Hofer S. J. Simon A. K. Bergmann M. Eisenberg T. Kroemer G. Madeo F. (2022). Mechanisms of spermidine-induced autophagy and geroprotection. Nat. Aging 2 (12), 1112–1129. 10.1038/s43587-022-00322-9 37118547

[B13] Hofsteen P. Robitaille A. M. Strash N. Palpant N. Moon R. T. Pabon L. (2018). ALPK2 promotes cardiogenesis in zebrafish and human pluripotent stem cells. iScience 2, 88–100. 10.1016/j.isci.2018.03.010 29888752 PMC5993047

[B14] Horvath S. (2013). DNA methylation age of human tissues and cell types. Genome Biol. 14 (10), R115. 10.1186/gb-2013-14-10-r115 24138928 PMC4015143

[B15] Horvath S. Oshima J. Martin G. M. Lu A. T. Quach A. Cohen H. (2018). Epigenetic clock for skin and blood cells applied to Hutchinson Gilford Progeria Syndrome and *ex vivo* studies. Aging (Albany NY) 10 (7), 1758–1775. 10.18632/aging.101508 30048243 PMC6075434

[B16] Jin P. Wu L. Zhang G. Yang B. Zhu B. (2022). PDZRN4 suppresses tumorigenesis and androgen therapy-resistance in prostate cancer. J. Cancer 13 (7), 2293–2300. 10.7150/jca.69269 35517421 PMC9066220

[B17] Kaluzinska-Kolat Z. Kosla K. Kolat D. Pluciennik E. Bednarek A. K. (2023). Antineoplastic nature of WWOX in glioblastoma is mainly a consequence of reduced cell viability and invasion. Biol. (Basel) 12 (3), 465. 10.3390/biology12030465 PMC10045224 36979157

[B18] Levine M. E. Lu A. T. Quach A. Chen B. H. Assimes T. L. Bandinelli S. (2018). An epigenetic biomarker of aging for lifespan and healthspan. Aging (Albany NY) 10 (4), 573–591. 10.18632/aging.101414 29676998 PMC5940111

[B19] Lewandowski N. M. Ju S. Verbitsky M. Ross B. Geddie M. L. Rockenstein E. (2010). Polyamine pathway contributes to the pathogenesis of Parkinson disease. Proc. Natl. Acad. Sci. U. S. A. 107 (39), 16970–16975. 10.1073/pnas.1011751107 20837543 PMC2947879

[B20] Liu Y. Su P. Zhao W. Li X. Yang X. Fan J. (2021). ZNF213 negatively controls triple negative breast cancer progression via Hippo/YAP signaling. Cancer Sci. 112 (7), 2714–2727. 10.1111/cas.14916 33939216 PMC8253295

[B21] Lopez-Otin C. Blasco M. A. Partridge L. Serrano M. Kroemer G. (2023). Hallmarks of aging: an expanding universe. Cell 186 (2), 243–278. 10.1016/j.cell.2022.11.001 36599349

[B22] Lowe R. Gemma C. Beyan H. Hawa M. I. Bazeos A. Leslie R. D. (2013). Buccals are likely to be a more informative surrogate tissue than blood for epigenome-wide association studies. Epigenetics 8 (4), 445–454. 10.4161/epi.24362 23538714 PMC3674053

[B23] Lu J. Zhao Q. Wang L. Li J. Wang H. Lv L. (2024). MBNL2 promotes aging-related cardiac fibrosis via inhibited SUMOylation of Kruppel-like factor4. iScience 27 (7), 110163. 10.1016/j.isci.2024.110163 38974966 PMC11226984

[B24] Lu Y. L. Yang X. Liu Y. K. (2019). Reduced PDZRN4 promotes breast cancer progression and predicts poor prognosis. Int. J. Clin. Exp. Pathol. 12 (1), 142–153. 31933728 PMC6944026

[B25] Lund J. B. Li S. Baumbach J. Svane A. M. Hjelmborg J. Christiansen L. (2019). DNA methylome profiling of all-cause mortality in comparison with age-associated methylation patterns. Clin. Epigenetics 11 (1), 23. 10.1186/s13148-019-0622-4 30736859 PMC6368749

[B26] Moverare-Skrtic S. Voelkl J. Nilsson K. H. Nethander M. Luong T. T. D. Alesutan I. (2023). B4GALNT3 regulates glycosylation of sclerostin and bone mass. EBioMedicine 91, 104546. 10.1016/j.ebiom.2023.104546 37023531 PMC10102813

[B27] Oughtred R. Rust J. Chang C. Breitkreutz B. J. Stark C. Willems A. (2021). The BioGRID database: a comprehensive biomedical resource of curated protein, genetic, and chemical interactions. Protein Sci. 30 (1), 187–200. 10.1002/pro.3978 33070389 PMC7737760

[B28] Pegg A. E. (2008). Spermidine/spermine-N(1)-acetyltransferase: a key metabolic regulator. Am. J. Physiol. Endocrinol. Metab. 294 (6), E995–E1010. 10.1152/ajpendo.90217.2008 18349109

[B29] Shin B. C. Cepeda C. Estrada-Sanchez A. M. Levine M. S. Hodaei L. Dai Y. (2018). Neural deletion of glucose transporter isoform 3 creates distinct postnatal and adult neurobehavioral phenotypes. J. Neurosci. 38 (44), 9579–9599. 10.1523/JNEUROSCI.0503-18.2018 30232223 PMC6706001

[B30] Shokhirev M. N. Torosin N. S. Kramer D. J. Johnson A. A. Cuellar T. L. (2024). CheekAge: a next-generation buccal epigenetic aging clock associated with lifestyle and health. Geroscience 46 (3), 3429–3443. 10.1007/s11357-024-01094-3 38441802 PMC11009193

[B31] Singh P. P. Demmitt B. A. Nath R. D. Brunet A. (2019). The genetics of aging: a vertebrate perspective. Cell 177 (1), 200–220. 10.1016/j.cell.2019.02.038 30901541 PMC7592626

[B32] Slieker R. C. Relton C. L. Gaunt T. R. Slagboom P. E. Heijmans B. T. (2018). Age-related DNA methylation changes are tissue-specific with ELOVL2 promoter methylation as exception. Epigenetics Chromatin 11 (1), 25. 10.1186/s13072-018-0191-3 29848354 PMC5975493

[B33] Stevenson A. J. McCartney D. L. Hillary R. F. Redmond P. Taylor A. M. Zhang Q. (2019). Childhood intelligence attenuates the association between biological ageing and health outcomes in later life. Transl. Psychiatry 9 (1), 323. 10.1038/s41398-019-0657-5 31780646 PMC6883059

[B34] Suzuki S. Ando F. Kitagawa S. Hara Y. Fujiki T. Mandai S. (2023). ZNF185 prevents stress fiber formation through the inhibition of RhoA in endothelial cells. Commun. Biol. 6 (1), 29. 10.1038/s42003-023-04416-x 36631535 PMC9834212

[B35] Taylor A. M. Pattie A. Deary I. J. (2018). Cohort profile update: the lothian Birth cohorts of 1921 and 1936. Int. J. Epidemiol. 47 (4), 1042–1042r. 10.1093/ije/dyy022 29546429 PMC6124629

[B36] Thrush K. L. Higgins-Chen A. T. Liu Z. Levine M. E. (2022). R methylCIPHER: a methylation clock investigational package for hypothesis-driven evaluation and research. bioRxiv 2022, 499978. 10.1101/2022.07.13.499978

[B37] Wang Y. Wu J. Luo W. Zhang H. Shi G. Shen Y. (2021). ALPK2 acts as tumor promotor in development of bladder cancer through targeting DEPDC1A. Cell Death Dis. 12 (7), 661. 10.1038/s41419-021-03947-7 34210956 PMC8249393

[B38] Xiong Z. Li M. Yang F. Ma Y. Sang J. Li R. (2020). EWAS Data Hub: a resource of DNA methylation array data and metadata. Nucleic Acids Res. 48 (D1), D890–D895. 10.1093/nar/gkz840 31584095 PMC6943079

[B39] Zang X. Wang Q. Zhang H. Zhang Y. Wang Z. Wu Z. (2024). Knockdown of neuronal DAF-15/Raptor promotes healthy aging in *C. elegans* . J. Genet. Genomics 51 (5), 507–516. 10.1016/j.jgg.2023.11.002 37951302

[B40] Zhang Q. Vallerga C. L. Walker R. M. Lin T. Henders A. K. Montgomery G. W. (2019). Improved precision of epigenetic clock estimates across tissues and its implication for biological ageing. Genome Med. 11 (1), 54. 10.1186/s13073-019-0667-1 31443728 PMC6708158

[B41] Zheng S. C. Breeze C. E. Beck S. Dong D. Zhu T. Ma L. (2019). EpiDISH web server: epigenetic dissection of intra-sample-heterogeneity with online GUI. Bioinformatics 36 (6), 1950–1951. 10.1093/bioinformatics/btz833 31710662 PMC7703755

[B42] Ziegler A. Duclaux-Loras R. Revenu C. Charbit-Henrion F. Begue B. Duroure K. (2021). Bi-allelic variants in IPO8 cause a connective tissue disorder associated with cardiovascular defects, skeletal abnormalities, and immune dysregulation. Am. J. Hum. Genet. 108 (6), 1126–1137. 10.1016/j.ajhg.2021.04.020 34010604 PMC8206386

