# Supplementary material for: CheekAge, a next-generation epigenetic buccal clock, is predictive of mortality in human blood
Source: Front Aging. 2024 Oct 1;5:1460360. doi: 10.3389/fragi.2024.1460360 (PMC11473594; doi:10.3389/fragi.2024.1460360)
Supplement: Supplementary file 2 [file Presentation1.pdf]

Supplementary Figure S1. Distribution of mortality and anti-mortality CpGs. With the CpG index (sorted by FDR) on the x-axis and the -log(FDR) of the overall mortality association post-CpG removal on the y-axis, the distribution of CpG-level effects is visualized. DNA methylation sites that improve or worsen the mortality association when removed were dubbed anti-mortality CpGs or mortality CpGs, respectively.

Supplementary Figure S2. Enrichment results for the top 250 annotated mortality CpGs. For the top 250 mortality CpGs with gene annotations, a network topology-based enrichment analysis was performed using WebGestalt. The top 15 Gene Ontology processes are visualized and sorted by -log(FDR).

Supplementary Figure S3. Enrichment results for the top 250 annotated anti-mortality CpGs. For the top 250 anti-mortality CpGs with gene annotations, a network topology-based enrichment analysis was performed using WebGestalt. The top 15 Gene Ontology processes are visualized and sorted by -log(FDR).
