# Supplementary figures and images for: CheekAge, a next-generation epigenetic buccal clock, is predictive of mortality in human blood
Source: Front Aging. 2024 Oct 1;5:1460360. doi: 10.3389/fragi.2024.1460360 (PMC11473594; doi:10.3389/fragi.2024.1460360)

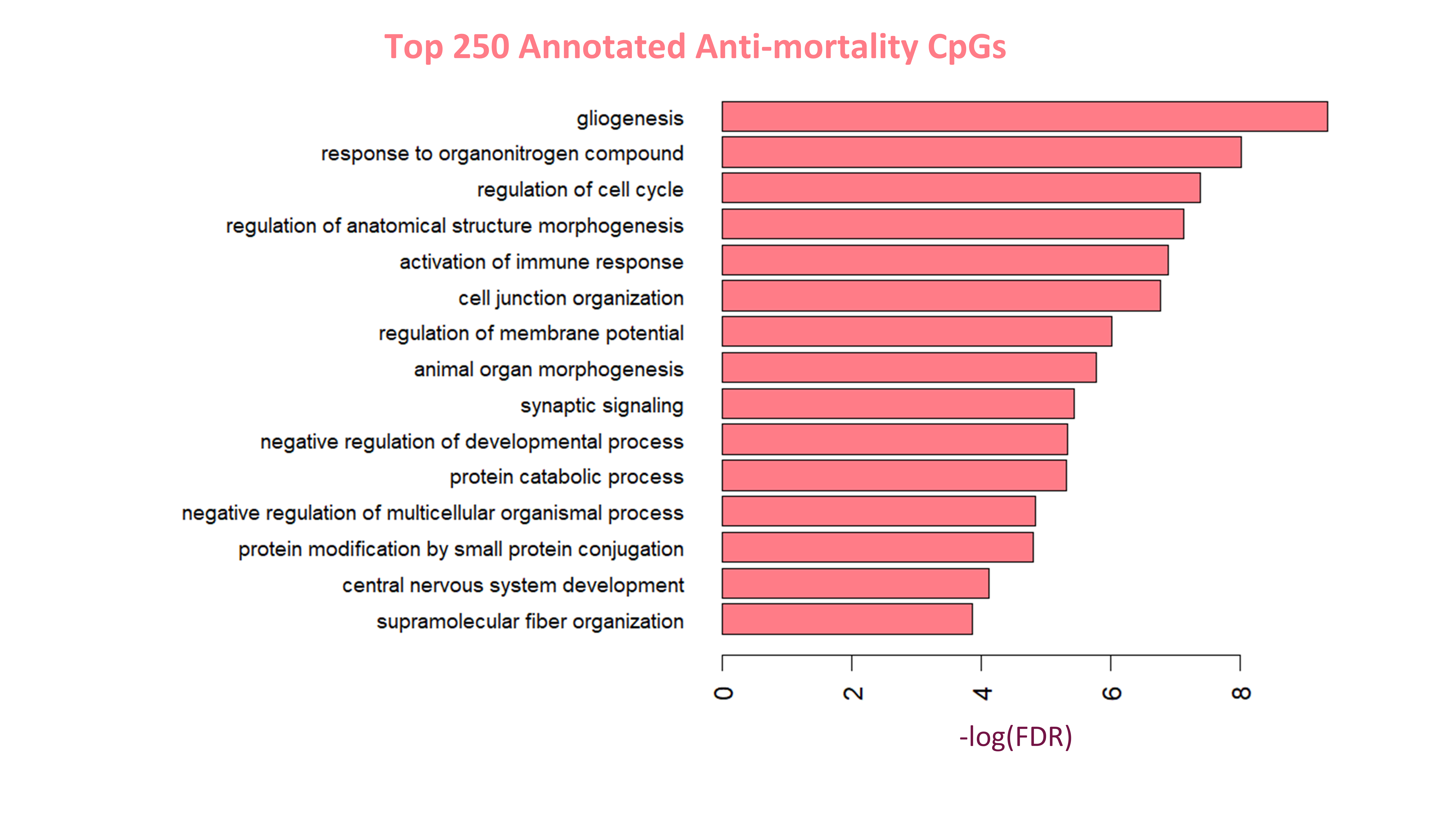

Supplement: Supplementary file 3 [file Image3.tif]

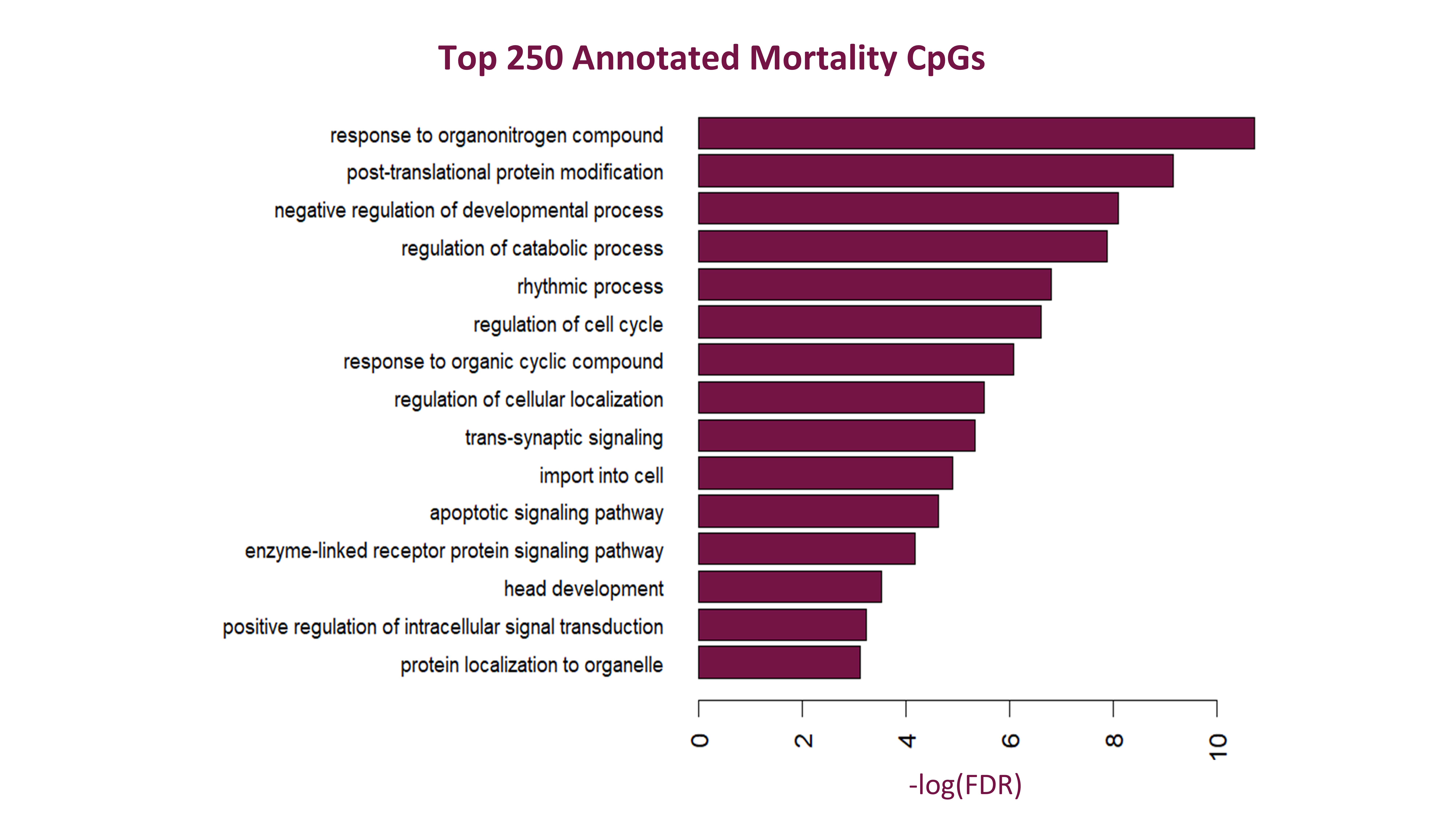

Supplement: Supplementary file 4 [file Image2.tif]

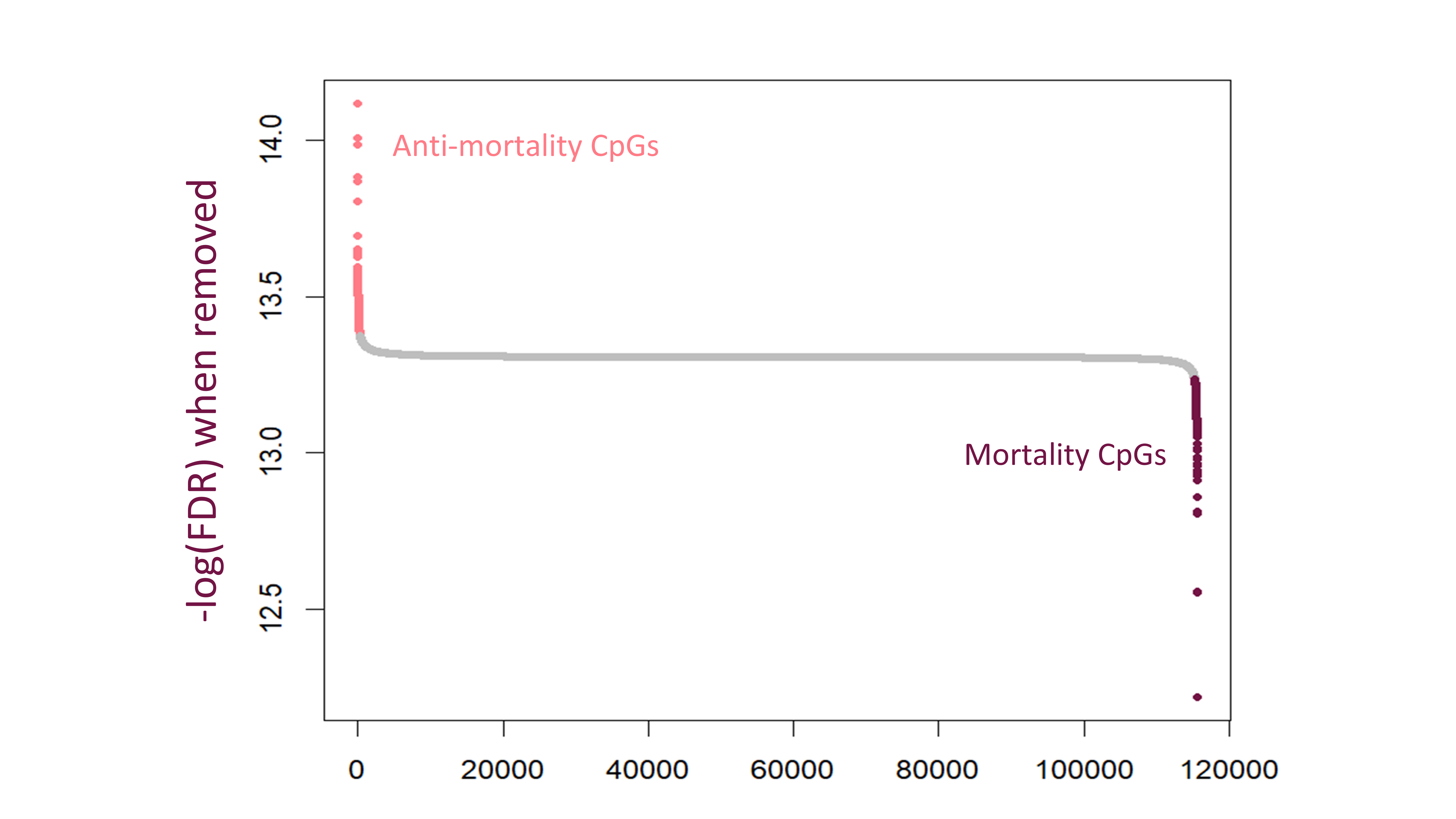

Supplement: Supplementary file 5 [file Image1.tif]
